# Candidate neuroinflammatory markers of cerebral autoregulation dysfunction in human acute brain injury

**DOI:** 10.1177/0271678X231171991

**Published:** 2023-05-03

**Authors:** Claudia A Smith, Keri LH Carpenter, Peter J Hutchinson, Peter Smielewski, Adel Helmy

**Affiliations:** Division of Neurosurgery, Department of Clinical Neurosciences, 2152 University of Cambridge, Cambridge, UK

**Keywords:** Cerebral autoregulation, cerebral blood flow, biomarkers, brain trauma, inflammation

## Abstract

The loss of cerebral autoregulation (CA) is a common and detrimental secondary injury mechanism following acute brain injury and has been associated with worse morbidity and mortality. However patient outcomes have not as yet been conclusively proven to have improved as a result of CA-directed therapy. While CA monitoring has been used to modify CPP targets, this approach cannot work if the impairment of CA is not simply related to CPP but involves other underlying mechanisms and triggers, which at present are largely unknown. Neuroinflammation, particularly inflammation affecting the cerebral vasculature, is an important cascade that occurs following acute injury. We hypothesise that disturbances to the cerebral vasculature can affect the regulation of CBF, and hence the vascular inflammatory pathways could be a putative mechanism that causes CA dysfunction. This review provides a brief overview of CA, and its impairment following brain injury. We discuss candidate vascular and endothelial markers and what is known about their link to disturbance of the CBF and autoregulation. We focus on human traumatic brain injury (TBI) and subarachnoid haemorrhage (SAH), with supporting evidence from animal work and applicability to wider neurologic diseases.

## Introduction

Traumatic brain injury (TBI) and subarachnoid haemorrhage (SAH) are often grouped together as ‘Acute Brain Injuries’ and are responsible for a substantial burden of neurological morbidity and mortality. There are many factors influencing outcome after brain injury and these processes are dynamically changing over the first few days post-injury. Clinical management of patients is focussed on the prevention of secondary brain injury and on providing a physiologically stable environment for brain recovery. One common contribution to secondary brain injury in both TBI and SAH is the loss of cerebral autoregulation (CA).

CA is the brain’s physiological mechanism that stabilises cerebral blood flow (CBF) and ensures that the brain is supplied with adequate nutrients and oxygen despite changes in cerebral perfusion pressure (CPP, the mathematical difference between mean arterial pressure [MAP] – intracranial pressure [ICP]).^1,2^ In severe brain trauma, fully functional CA guards against derangement in CPP, thereby avoiding ischaemia and hyper-perfusion.^3,4^ On the other hand, impaired CA has been associated with worsened outcome and mortality in severe TBI^5,6^ and is understood to be a key factor in the development of secondary injury. Despite developments made in TBI injury dynamic CA monitoring, mortality and outcome seem to have remained relatively unchanged in the last two decades.^7^ This may, at least partially, reflect a lack of understanding of the molecular mechanistic underpinnings of CA, and therefore a lack of means of therapeutically modifying CA disturbance. Investigations into the possible mechanisms causing CA impairment is fundamental in directing and developing potential patient therapies and improving overall outcome following injury.

It is widely accepted that neuroinflammation plays a role in brain injury.^8–10^ In the initial period after injury, the pro-inflammatory response aids in the preservation of surviving neuronal tissue and cell repair.^11,12^ However, if the inflammatory response is chronic as it is in brain injury, it may be detrimental and harmful to recovery.^13^ The neuroinflammatory cascade inflicts direct injury to neurons and supporting cells, but also includes mediators, cytokines and chemokines that set off other potentially detrimental cascades, some or all of which may contribute to secondary brain injury. One such continuous and resultant cascade is vascular inflammation. This may cause and exacerbate vascular pathological states, such as endothelial cell swelling/oedema^14^ within cerebral blood vessel walls and increased vascular permeability. These changes result in decreased vascular reactivity and can contribute to the inadequate CBF following brain injury. Neuroinflammation affecting the vasculature provides a promising putative link between the inflammatory pathway and CA disturbances following TBI and SAH.

In this review, we give a brief overview of CA, and what is known regarding its impairment following acute brain injury. We add supporting evidence from animal work where relevant, but mainly focus on human TBI and SAH. Then, we discuss candidate vascular and endothelial markers and what is known about their link to disturbance of the CBF and autoregulation. We acknowledge and recognise the applicability of this work to other neurologic diseases, and briefly discuss the advantage of future work directed at the molecular underpinning of CA dysfunction to multiple fields.

## Brief overview of cerebral autoregulation

CA is an important physiological mechanism that ensures adequate CBF despite changes in CPP. If CA is impaired, CBF will fluctuate passively with CPP which puts the already-injured brain at risk for further secondary injury. If CPP, and therefore CBF if CA is impaired, is too low, the brain is at risk of ischaemia; if CPP is too high, there is a risk of aggravated brain oedema resulting in a secondary rise of ICP.

As this is such an important contribution to brain homeostasis, there are several mechanisms by which vascular tone within blood vessels and therefore their calibre is regulated: myogenic, metabolic (including biochemical) and neurogenic. There is significant overlap between these mechanisms, and the contribution of each mechanism is largely unknown.

### Genetic variation affecting multiple mechanisms of CBF control

While it is generally accepted that the underlying mechanisms of autoregulation are largely unknown, there are existing postulations and evidence for some mechanisms in the literature. The genetic drivers and polymorphisms that may play an important role in CA following TBI have been discussed in the review by Zeiler et al.^15^ This synopsis of investigations of CA mechanisms from a genetic standpoint have highlighted pathways involving inflammation, central autonomic response, and cortical spreading depression. Single-nucleotide polymorphisms (SNPs) relating to endothelial, myogenic, neurotransmitters and metabolic mechanisms in the brain vasculature were identified as contributing to CA. Additionally, the renin-angiotensin system has been involved in modulation of the vascular myogenic responses, with SNPs in genes encoding angiotensin converting enzyme (ACE) and the type 2 angiotensin II receptor (AT2) linking to the development of delayed ischaemic neurological deficits in SAH.^16,17^ Furthermore, the recessive allele of ACE has been linked to worse outcome scores at 6 months post TBI.^15,18^ Identifying candidate SNPs may be useful as key pathways are highlighted, but the manifestation of these key genetic pathways in the clinical setting is highly influenced by multiple factors. While genetic investigations are important for identifying fundamental predispositions to CA dysfunction, it is necessary to study CBF control in the clinical setting.

### Myogenic

**Figure 1.**
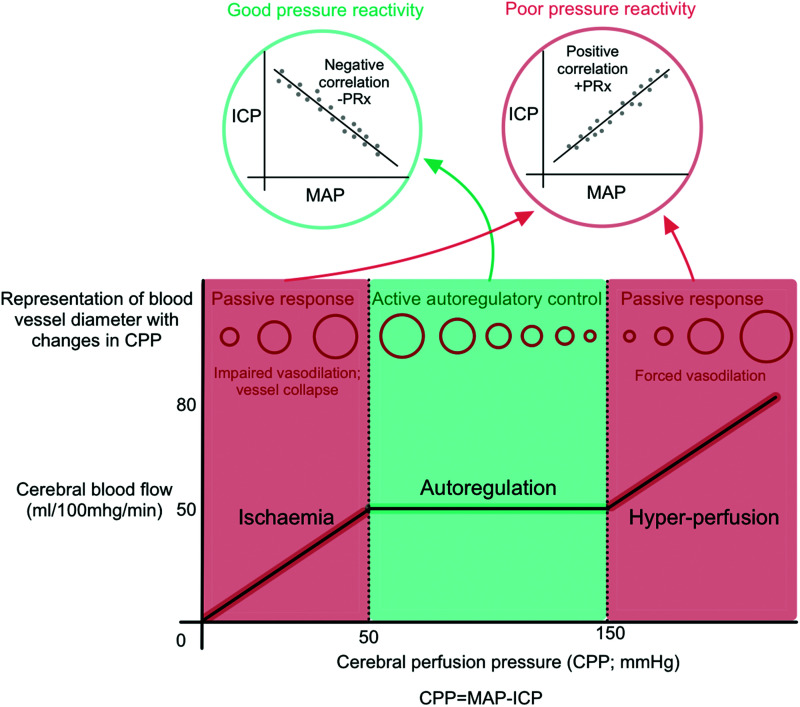
An overview of cerebral autoregulation. Cerebral autoregulation functions over a range of cerebral perfusion pressures (CPP; the mathematical difference between mean arterial pressure [MAP] and intracranial pressure [ICP]). Blood pressures outside of the autoregulatory zone cause passive changes in cerebral blood flow and puts the patient at risk of secondary injury. One autoregulatory mechanism of cerebral blood flow control is cerebrovascular pressure reactivity. The pressure reactivity index (PRx) is the correlation coefficient between the MAP and ICP, with a negative PRx indicating good pressure reactivity.

The myogenic mechanism of CA is based on the intrinsic ability of smooth muscle cells in the cerebral blood vessels to constrict and dilate in response to changes in transmural pressure.^19^ PRx, the pressure reactivity index, is a mathematical indicator of cerebrovascular pressure reactivity and is calculated as the correlation coefficient between MAP and ICP. PRx is calculated as the moving correlation coefficient between MAP and ICP, and ranges from −1 to 1, with negative values suggesting intact pressure reactivity, and positive values indicating impaired reactivity.^20^ Its main assumption is that ICP reflects cerebrovascular volume changes brought about by slow variations in MAP (Figure 1). It has been shown that PRx has a strong and independent association with clinical outcome.^4,21^

The myogenic mechanism of CA is supported by primary observations of cerebral arteries changing diameter in response to blood pressure stimuli. These reactions persist when neurogenic control of the vessels is eliminated.^19,22–25^ Changes in transmural pressure and blood flow induce diameter changes in cerebral blood vessels, however the exact molecular mechanisms acting on the endothelium are largely unknown.

Calcium signalling, and fluxes in ion concentration is fundamental to the functioning of most cells and plays an important role in the initiation of the myogenic response of CA. Calcium flux in the smooth muscle cells of cerebral vessels lead to eventual vasoconstriction as follows: changes in transmural pressure and wall tension are detected by a mechanoreceptor, which leads to the opening of voltage gated calcium channels. Calcium floods the cell and activates myosin light chain kinase (MLCK).^26^ This leads to myosin phosphorylation and actin polymerisation, and smooth muscle cell contraction. Alternatively, rapid dephosphorylation of the myosin heavy chain promotes relaxation.^27^

Importantly, while calcium influx to the cell is required for the myogenic response, voltage gated calcium channels is not the only way to increase intracellular calcium. Activation of membrane-bound phospholipase C leads to the production of 1,4,5-triphosphate (IP3) and 1,2 diacylglycerol (DAG). IP3 will then diffuse into the cell and stimulate the sarcoplasmic reticulum to release calcium.^27,28^

### Metabolic

The metabolic mechanism of CA is the mechanism that links the local rate of metabolism to cerebral vasculature, hence controlling CBF. The brain’s energy demand is tightly linked to the blood supply of oxygen and nutrients.^29^ Therefore, this mechanism is stimulated due to changes in the local metabolism in the cerebral vasculature, such as cerebral hypercapnia. The brain vasculature is highly sensitive to changes in partial pressure of arterial carbon dioxide (PaCO_2_).^30^ A high PaCO_2_ will cause vasodilation, resulting in an increase of CBF. This mechanism serves as an automatic feedback loop – increased brain activity and metabolism will lead to an increase in PaCO_2_, as detected by surrounding neurons. These neurons then produce neuronal nitric oxide synthase (nNOS) which subsequently facilitates vasodilation, and an increase in CBF.^31–33^ Endothelial-derived NOS (eNOS) also leads to vasodilation (to be discussed later) but the hypercapnia-induced vasodilation seems to be nNOS-dependent.^34,35^

Importantly, this metabolic mechanism (CO_2_ reactivity and flow-metabolism coupling) is different from pressure reactivity as it is, largely, CPP-independent.^36^ Another important distinction of note is between the metabolic mechanism of CA, and neurovascular coupling. The latter is a mechanism by which CBF is regulated according to local cortical/neuronal activity.^29^ The intuitive concept that neuronal substrate demand via increased activity (i.e., increased metabolic demand) is linked to supply (blood flow) is oversimplified, and it is likely that neuronal signalling itself (likely via neurotransmitters and/or extracellular potassium ions) are involved in this mechanism.^37,38^ There is definite overlap and interaction between these two important CBF regulatory mechanisms, but these terms should not be used interchangeably.

### Neurogenic

The neurogenic mechanism of CA refers to the direct control of cerebral vasculature via autonomic innervation. Intrinsic/central innervation of cerebral vessels is likely to have a global effect on CBF control, with modulations from extrinsic innervation (in addition to inflammatory, metabolic, and myogenic influences). Cerebral arteries are extensively innervated, and have been shown to constrict following adrenergic stimulation, and dilate following cholinergic stimulation. However, sympathetic and parasympathetic denervation did not abolish CBF regulation or reactivity to changes in PaCO_2._^39,40^ This implies that the neurogenic mechanism can modulate CBF control, but it is not a primary CA control mechanism. Interestingly, calcium signalling, as in the myogenic response, is also important for neurovascular coupling at the capillary level specifically, as astrocytic calcium is necessary for vasodilation of capillaries.^27,41^

An important note in the investigations of CBF regulation is the brain’s redundancy in maintain overall homeostasis, with adequate blood flow supply being no exception.^42^ Overlapping mechanisms contribute to maintain CBF under challenging circumstances. Therefore, another research challenge when studying CBF control is the overlap and interplay of all mechanisms, and the overall effects on pathway derangements in diseased states. It is very likely that multiple mechanisms act simultaneously and together. It is generally accepted that the myogenic and metabolic mechanisms control CBF and thereby maintain CA homeostasis, which is further modified by the neurogenic mechanism. The molecular processes underlying these mechanisms, and therefore CA, is largely unknown. However, key pathways in endothelial dysfunction and vascular inflammation have been identified, and potential candidates along these pathways will be discussed below.

## Endothelial dysfunction and vascular inflammation: vasoactive substances in brain injury

Endothelial dysfunction is characterized by a predominance of a more pro-inflammatory state and reduced vasodilation, and hence reduced CBF.^43^ This dysfunction of the endothelial cells in cerebral blood vessels occurs commonly after brain injury, and often leads to decreased CBF to the already ischaemic brain.^44^

**Figure 2.**
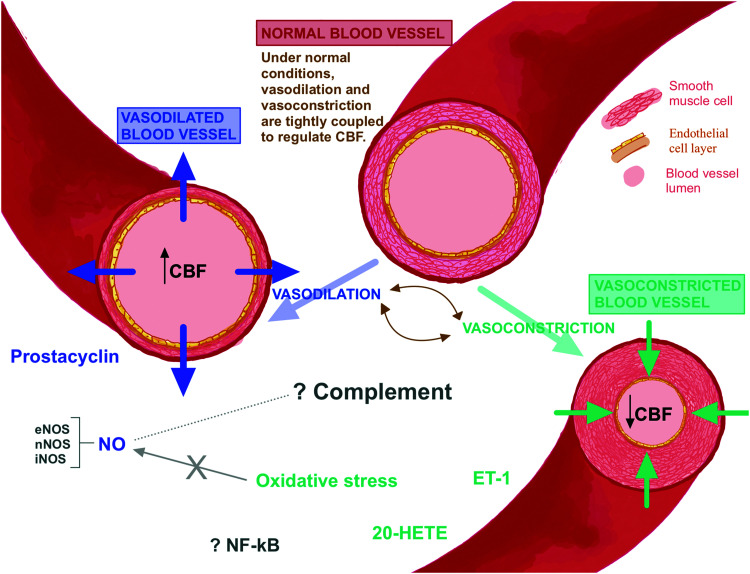
Schematic figure (not to scale): under normal healthy conditions, constriction and dilation of the blood vessel are tightly regulated to maintain a constant cerebral blood flow (CBF). Nitric oxide (NO) and prostacyclin induce vasodilation which results in increased cerebral blood volume (CBV). On the other hand, endothelin-1 (ET-1), 20-hydroxyeicosatrienoic-acid (20-HETE), and oxidative stress induce vasoconstriction, which leads to increased CBV. In pathologies such as TBI and SAH, these vasoactive substances that influence vasoconstriction and vasodilation can be up- or down-regulated, and hence CBF may be impacted. The influence of the complement system, nuclear factor kappa B (NF-kB) and the interplay between all the vasoactive substances is largely unknown in brain injury.

Endothelial derived vasoactive substances may provide a promising link between neuroinflammation and CA autoregulation disturbances, particularly cerebrovascular pressure reactivity impairment. Figure 2 provides a summary of the various potential pathways involved in CBF control. Importantly, these pathways are complex, and the mediators mentioned below often act in an environmentally dependent fashion, where specific fine-tuning of the cellular environment can alter the function of the mediator. Here, we discuss candidate mediators while acknowledging the complexities and intricacies involved in studying their functions and actions in health and disease.

### Nitric oxide (NO)

Nitric oxide (NO) is the archetypal vasodilator that acts on the smooth muscle cells of cerebral arterial walls. NO is synthesized from the amino acid L-arginine by nitric oxide synthase (NOS), a membrane bound enzyme. Release of NO results in cytosolic calcium release, and causes relaxation of smooth muscle fibres, thereby resulting in vasodilation and increased CBF.^45,46^ Apart from its vasodilatory function, NO also reduces vascular permeability, platelet aggregation, tissue oxidation and inflammation, cell growth and proliferation and migration.^46^ However, there is also literature to suggest NO results in increased blood brain barrier (BBB) permeability.^47^ Contradictory findings in the literature^48,49^ highlight the complexity of NO signalling, and the difficulty in characterising the exact effects of this molecular pathway.

There are three isoforms of NOS: endothelial (eNOS), neuronal (nNOS), and inducible (iNOS, Table 1). nNOS, and eNOS are expressed constitutively in healthy conditions and its production is calcium-dependent, while iNOS is not expressed unless induced by pro-inflammatory cytokines or endotoxins, often in pathology. iNOS production is calcium-independent and is therefore present 100–1000 fold higher than the other NOS-derived isoforms.^50,51^ In healthy conditions, CBF is likely controlled via the nNOS and eNOS isoform-derived NO pathways. However, in TBI and SAH there is the conceivable added effect of iNOS on CBF control.

**Table 1.** A summary of the three different isoforms of NOS, and main functions of the NO derived from this isoform of NOS.

Isoform of NOS^a^	Where it is expressed^51^	Main functions of NOS-derived NO
eNOS	Vascular endotheliumChoroid plexus	Regulation of vascular hemodynamics^51,154–156^Preserving and maintaining the cerebral microcirculation^156^Inhibiting platelet aggregation, leukocyte adhesion and migration^157^Reducing smooth muscle proliferation^156^
nNOS	Neuronal cell bodies	Neurovascular coupling^51,156,158,159^Neuronal plasticity^160^Memory formation^160^Regulation of CBF^161^Transmission of pain signals^162^Neurotransmitter release^161^
iNOS	MacrophagesGlial cellsVascular smooth muscle and endothelial cellsTumour cellsChoroid plexus	Inflammation^51,156,163^

^a^We refer interested readers to the Garry et al.^51^ review that discusses the various roles and functions of the NOS isoforms, and to that of Toda et al.^156^ that summarises NO and CBF regulation.

NO has multiple complex roles in normal physiological conditions, and therefore its effects may conceivably be both advantageous and detrimental in pathological states. In traumatic brain injury (TBI), NO accumulates in the brain immediately after injury, seems to be rapidly depleted 30 minutes to 6 hours post-injury, and then gradually accumulates again after 6 hours.^52^ Experimental models have demonstrated increased NO concentrations immediately after trauma, measured directly with a NO electrode and indirectly via microdialysate concentrations.^53,54^ This initial peak is likely the effect of upregulated eNOS and nNOS as iNOS was undetectable.^55^ Further exploration using electron paramagnetic resonance spectroscopy in mice brains showed that 90% of the accumulating NO in the brain within the first 10 to 20 minutes after the onset of global cerebral ischaemia was produced by nNOS, while the other 10% was produced via the eNOS. NO produced by nNOS during this time is likely to have adverse effects on outcome, whereas eNOS-produced NO is likely to play an advantageous role by preserving CBF.^52^ This dual effect of NO has been seen in ischaemia models,^49,56^ and studies using NOS inhibitors,^52,54^ strongly suggesting this action of NO following trauma. As summarised by Cherian et al., the administration of general NOS inhibitors (i.e., not specifically targeting any isoform of NOS) found mixed effects on outcome following trauma in the animal models, however specific inhibition of nNOS is found to be protective, thereby suggesting a detrimental action of nNOS^52^ in this initial time-period.

Relative depletion of NO after this initial peak follows for a combination of reasons. Firstly, NOS gene expression can be impaired after trauma, and decreased production of NOS could be because of this oxidative DNA damage. This can recover with time. Additionally, NOS requires 3 co-substrates: L-arginine, oxygen, and nicotinamide adenine dinucleotide phosphate (NADPH). Following trauma, a lack of these necessary substrates is likely to impair NO synthesis. Finally, there could be inhibition of NOS, and inactivation of NOS by free radicals (e.g. superoxide) following trauma. This overall decreased bioavailability of NO (increased metabolism of NO, lack of NOS or likely both) puts the brain at risk of decreased CBF and ischaemia. After 6 hours, NO gradually accumulates and stabilizes. This is likely to be because of iNOS production, which may account for general increase in NO levels during this ‘late’ phase. However, there is some conflicting evidence of the role of iNOS following acute brain injury – whether it is detrimental or neuroprotective following trauma is still unknown, but most models show that the inhibition of iNOS has advantageous effects.^52,57^

Villalba et al. used rat models to demonstrate that TBI causes increased NO production following injury via an upregulation of iNOS in endothelial cells.^58^ This led to enhanced vasodilation and the loss of myogenic tone. The authors postulate that defective myogenic tone may be contributing to the loss of CA following TBI but recognise that it is unclear whether enhanced vasodilation is a protective response, or a deleterious contributor to secondary injury following injury.

NO is a short-lived, highly reactive species but its major products nitrate and nitrite (collectively termed NOx) can readily be measured retrospectively in biological samples as markers of nitric oxide production. A pilot study by Carpenter et al. of 12 patients (11 TBI, one SAH) receiving neurocritical care has shown that as microdialysate NOx increases, glucose increases, lactate decreases, and the L/P ratio decreases.^59^ These findings suggest that in human acute brain injury, NO may improve blood supply, delivering more oxygen and glucose to brain tissue, and shifting metabolism towards the TCA cycle. The general temporal pattern was that NOx concentrations within-patient were relatively higher at earlier time-points compared with later time-points,^59^ consistent with the findings of Hlatky et al. in TBI,^60^ and of Sakowitz et al. in SAH.^61^ In the SAH patients studied by Carpenter et al., microdialysis started 2 days post-SAH and continued for 10 days, with NOx concentrations measured in the final 2 days, during which time fluctuations in NOx were followed by a decline to markedly low levels, with unfavourable clinical outcome.

SAH patients experience a decrease in CBF, which may be further exacerbated by NO depletion. NO signalling pathways have been shown to play a significant role in the risk of delayed cerebral ischaemia following SAH. This is of particular importance for the eNOS gene, as some genetic variants influences the patient’s risk of DCI following SAH as lower eNOS activity results in increased risk,^62^ showing the protective effect of eNOS in SAH, as in TBI.

Additionally, the functional uncoupling of eNOS from its co-factor tetrahydrobiopterin (BH4) during cerebral ischaemia, and results in the production of superoxide instead of NO.^63^ Superoxide then goes on to react with NO to form peroxynitrite – this is detrimental as it not only decreases bioavailability of NO, but peroxynitrite is neurotoxic itself,^51,64,65^ causing mitochondrial damage, vascular endothelial and smooth muscle cell damage.^66^

In TBI and SAH, increased oxidative stress is a significant contributor to the development of secondary injury, and exacerbation of pathology.^67,68^ Multiple sources of excessive free radical generation include disrupted metabolism and mitochondrial functioning, as well as upregulation of enzymes that produce free radicals, and downregulation of enzymes involved in antioxidant systems. Reactive oxygen species can lead to smooth muscle cell hypertrophy and endothelial apoptosis, which may contribute to dysfunctional CA and CBF maintenance.^69^ Oxidative stress has been consistently associated with reduced NO function, and hence reduced endothelium-dependent relaxations. Reactive oxygen species inhibit the vasodilatory functions of NO,^45^ causing further decrease in CBF following acute brain injury.

While there is a large body of evidence to implicate the NOS/NO pathway in CA and CBF control after TBI, there is conflicting evidence about the role that each isoform of NOS has to play. Theoretically, it seems that that an upregulation of eNOS, and downregulation of nNOS and iNOS could preserve CBF after injury, and possibly avoid exacerbation of secondary ischaemia.

### Prostaglandins

Prostaglandins are a group of lipids that have both homeostatic and pathological functions. Like many inflammatory mediators, prostaglandins can be beneficial and deleterious following injury, but they act primarily as powerful vasodilators (although some animal work suggests prostaglandins may have vasoconstrictory properties as well^70–72^). These lipids act locally on the smooth muscle cells in the cerebral blood vessels, and also play a role in blood vessel permeability (as pro-inflammatory production of prostaglandins facilitate leukocyte movement from blood vessels to injured tissue). Therefore, it is unsurprising that prostaglandin changes may alter CBF and vascular reactivity. Prostacyclin, an organic compound in the prostaglandin family, has been implicated in mediating autoregulatory responses as it is produced in the walls of cerebral vessels. It not only acts as a potent vasodilator, but also inhibits platelet aggregation and is reduced in the acute period following SAH.^73,74^ This suggests that its function as a regulator of CBF may be impaired in SAH, implicating this compound’s mechanistic role in CA.

### Endothelin-1

Endothelial cells are stimulated to produce endothelin-1 (ET-1) by ischaemic insult and shear stress,^75^ causing vasoconstriction. In normal conditions, ET-1 release is tightly controlled and balanced with vasodilatory modulators, and the balance between vasoconstrictors (such as ET-1) and vasodilators (mainly NO) is vital in controlling CBF and endothelial function.^76^ However, after brain injury, this balance is disrupted. SAH patients not only have an upregulation of ET-1,^77^ but increased expression of the ET-1 receptor on the endothelium of cerebral arteries.^78,79^ Elevated levels of ET-1 are also seen following TBI^80^ as well as after acute ischaemic stroke^81,82^ where higher ET-1 serum concentrations were associated with more severe brain oedema.^83^ The upregulation of ET-1 after brain injury is associated with clinical vasospasm and secondary injury likely because of its efficiency and long-lasting vasoconstriction effects.^84^ The source of ET-1 over-production has been investigated, with active astrocytes and endothelial cells being key candidates.^85^ Theoretically, treatment aimed at decreasing ET-1 levels would be advantageous in increasing CBF and ameliorating some of ET-1’s harmful effects. A clinical trial in SAH patients looked at the effect of a selective ET-A receptor antagonist, clazosentan. The CONSCIOUS I-trial (Clazosentan to Overcome Neurological Ischaemia and Infarct OccUrring after Subarachnoid haemorrhage) showed a dose-dependent decrease in moderate to severe vasospasm, delayed ischaemic neurological deficits and new infarcts (seen on CT scan).^85,86^ This implicated ET-1 in the development of vasospasm in SAH. However, the CONSCIOUS II and III trials found no evidence that ET antagonists improved clinically relevant outcomes in SAH patients.^87,88^ This trial therefore provides evidence against a pure focus on macrovascular vasospasm as the cause of delayed ischaemia and resulting neurological deficit which is as expected: the development of vasospasm, just like the role of ET-1, is complex and involves several pathways, including microvascular thrombosis and brain injury induced at the time of ictus.

### 20-hydroxyeicosatrienoic-acid (20-HETE)

Vascular smooth muscle cells in cerebral artery walls express cytochrome P enzymes that result in the production of 20-hydroxyeicosatrienoic-acid (20-HETE), and that 20-HETE levels increase in response to elevations in intravascular pressure.^89^ 20-HETE is a potent vasoconstrictor arachidonic acid that is known to play a role in microvasculature in cerebral circulation, CBF control, inflammatory reactions, and cell proliferation.^90–94^ Inhibiting 20-HETE has shown advantageous effects in animal models: attenuated 20-HETE levels reduce cerebrovascular damage and improve stoke outcomes after cerebral ischaemia.^90,95–97^ Thus, it has been suggested that the administration of such an inhibitor could be neuroprotective after TBI and SAH. Lu et al. showed that inhibiting 20-HETE via the administration of a 20-HETE inhibitor, HET0016, protected against brain oedema in a traumatic rat model. Inhibition of 20-HETE also decreased blood brain barrier permeability following trauma. The group suggest that the administration of HET0016 decreased oxidative stress following trauma, and that allowed for the preservation of tight junction proteins and BBB integrity.^98^

20-HETE has been implicated in the myogenic response and CBF control^89,99^ and increased 20-HETE has been associated with increased myogenic response and CA adaptation to hypertension.^100–102^ Additionally, it has been shown that NO inhibits the cytochrome P enzymes involved in 20-HETE production, and that inhibition of 20-HETE contributes to vasodilation via NO pathways.^103^ Hence, this is further evidence that the balance between vascular mediators that promote vasoconstriction and dilation is vital for CA and CBF maintenance. We can also note that vasodilation via NO pathways has both 20-HETE dependent and independent (via guanylate cyclase^47,104–107^) mechanisms.

### Nuclear factor kappa B (NF-kB)

Nuclear factor kappa B (NF-kB) is a transcription factor that regulates the immune response, particularly promotes the production of pro-inflammatory cytokines, chemokines and cell adhesion molecules as part of innate immunity. The function of NF-kB has been explored, with endothelial, glial, and neuronal activation found during cerebral ischaemia.^108–110^ The exact vascular function of NF-kB in in healthy conditions and following injury, is largely unknown. However, its activation during cerebral ischaemia and the fact that the addition of putative neuroprotective antioxidants inhibits this NF-kB activation (and consequently results in reduced ischaemic neuronal death) suggests that the role of NF-kB following brain injury is deleterious.^111^ Interestingly, the fact that antioxidants can be used as NF-kB inhibitors suggests that intermediates of the reactive oxygen pathway are involved in NF-kB activation, possibly via a secondary messenger system.

Endothelial activation of this transcription factor may initiate, and exacerbate the pro-inflammatory cascade involved in the immediate hours following acute brain injury, and hence may play a role in CBF regulation. However, as NF-kB is an inducible transcription factor, it will likely be involved in the upstream CA molecular mechanisms and may have a non-specific inflammatory role in CBF regulation.

### Complement system

The complement system is part of the innate immune system and is a key pathway contributing to neuroinflammation and secondary injury following TBI.^112–114^ The complement cascade is controlled by multiple mediators, and the lack of this control leads to exacerbated neuropathology after trauma.^115^

There is evidence that complement plays a crucial role in the activation of microglia in the early stages of the immune response, as microglia express receptors for various complement proteins, including C1q and C3 cleavage products.^116,117^ During pathology, the C1q protein promotes the shift of microglia phenotype to M1, the pro-inflammatory phenotype involved with clearing cellular debris and foreign antigens but can also be harmful to healthy cells and cause further damage.^118^ Complement proteins C3a and C5a bind to their receptors and induce neuronal and glial apoptosis, as well as increase BBB leakage.^119^ With BBB disruption, a further influx of complement proteins exacerbates the inflammatory response.^120^ The terminal complement complex (TCC) is a downstream protein indicative of persistent and irreversible complement activation, and this protein further promotes neuronal lysis. Uncontrollable activation of the complement system therefore results in oxidative stress and free radical production, cell signalling disruption and further cell damage and apoptosis, thereby contributing significantly to secondary insult following brain injury.

The complement system has three different activation pathways: the classical, the alternative, and the lectin pathway.^121^ All pathways are triggered by complement initiator proteins binding to antibodies on antigens, or damaged cells. The activation of all pathways can be triggered by foreign antigen cells, as well as damaged endogenous cells– pathogen-associated molecular patterns (PAMPs) and damage-associated molecular patterns (DAMPs) respectively. The pathways are activated by different initiator molecules, and have different effector molecules, but these pathways all converge in that they all form C3 and C5 convertase, and subsequently activate the terminal complement pathway.^119^

There is evidence to suggest that all three pathways are active after brain injury. The key role of specific pathways is somewhat unknown and can be difficult to study considering that the pathways are intertwined and converge at common proteins. Animal studies have attempted to investigate the role of the three activation pathways. Mice lacking fB, a fundamental mediator in the alternative pathway (and hence whose alternative pathways are non-functional) has reduced neuronal loss, upregulation of anti-apoptotic proteins, and down regulation of anti-apoptotic receptors.^122^ In a similar study, alternative pathway inhibition via fB also showed reduced neuronal loss, decreased inflammatory response, and upregulated neuroprotection pathways, however there was no improvement in neurological functioning in these mice models.^123,124^

Animal model studies of TBI have investigated mice deficient for C4 (a protein involved in both the classical and lectin pathways) and found reduced tissue damage and motor deficits compared to wild type mice after controlled cortical injury. A study found that early administration of C1 inhibitor (functionally inhibiting both the classical and lectin pathways) allowed for preserved cognitive functioning and smaller contusion size, whereas delayed administration led to a reduction in motor dysfunction but had no effect on cognition or contusion size.^125^ Attempts at isolating the effects of these pathways further have been made. Substantial involvement of the classical pathway in TBI pathology is questioned, as mice deficient for C1q (a classical initiator) showed similar neurological deficits and lesion sizes to controls.^126^

**Figure 3.**
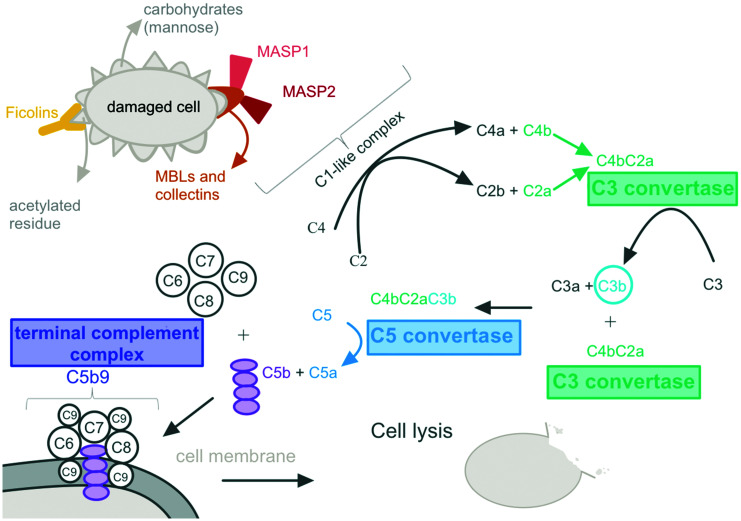
Evidence suggesting full and persistent activation of the lectin complement pathway after brain injury nominates potential candidate markers that could be associated with cerebral autoregulatory dysfunction.

There is a large body of evidence that implicates the lectin pathway in acquired brain injury, and hence potential analytes of this pathway could act as key biomarkers of CBF disturbance (Figure 3). Animal models have shown that activation of the lectin pathway early after TBI may have neuroprotective effects as mice without mannose binding lectin (MBL, an initiator protein) had greater neurological deficits and increased neurodegeneration in the early phase after cortical injury compared to controls.^127^ Notably, MBL has been found to be selectively deposited on the ischaemic endothelium as early as 30 minutes after focal cerebral ischaemia in mice.^128^ Five weeks post injury, mice models deficient in MBL had reduced neuronal loss,^129^ suggesting lectin pathway activity at the later stages after TBI are detrimental. The detrimental effects of lectin pathway activation are largely supported with evidence in cerebral ischaemia,^128^ in particular TBI,^130^ stroke,^131^ and SAH.^132^

A study by De Blasio et al. investigated lectin complement pathway activation in early (within 12 hours from injury) and late (12 hours to 5 days after injury) TBI patients. Immunofluorescence and confocal imaging analysis showed that lectin initiator molecules (MBL and ficolins), key enzymes (MBL-associated serine proteases, MASPs) and downstream components (C3 fragments and TCC) were elevated in TBI contusions compared to non-TBI contusions. These lectin complement proteins were found in the blood vessels and brain parenchyma of the TBI patients, and higher levels of MASP-2 were associated with altered pupil reactivity after traumatic SAH, and hence more severe clinical conditions.^133^ This finding is supported,^113,130,134^ with Burk et al. showing that serum levels of C3a and C5a of polytrauma patients correlate with injury severity and survival. This group further suggested that complement is rapidly activated and dysfunctional, thereby resulting in “complementopathy” after trauma.^113^

Lectin complement is not unique to TBI, however, De Blasio et al.’s evidence suggests that TBI favours full and persistent activation of the complement system, and its localisation to blood vessels as well as brain tissue of contusions suggests its contribution to secondary brain injury in acute brain damage pathophysiology. This study concluded that this complement activation depends on the lectin activation pathway with possible amplification by the alternative pathway; this activation is persistent up until 5 days post injury and is associated with injury severity.^133^

## Measuring these candidate markers

An ideal biomarker of CA ‘health’ would be a specific inflammatory marker that would be associated with the status of CA (i.e., reflecting whether CA is globally intact or impaired) of the patient, thus informing if CPP management efforts are at all likely to achieve improvement in CA. This candidate biomarker would need to be easily measured, and the levels rapidly quantified.

Inherent properties of the candidate marker would need to be considered. The molecular weight, half-life of the candidate marker, its stability and degradation at room temperature, and how the sample processing would affect the marker would affect the method of analysis and measurement. This is of particular concern for the NO pathway. NO production from the individual isoforms of NO synthase are difficult to study and there is currently no method to measure individual isoform-derived NO in human samples. Studies to date have looked at the overall NO levels via an invasive NO sensor (such as NO-selective electrodes used in-vivo in animal studies) or have studied NO metabolism products – nitrite and nitrate (measured ex-vivo in biofluids from patients or animals). Both methods do not give an indication of the source of the NO. Therefore, the study of NOS isoforms has come from animal models, via the use of specific NOS isoform inhibitors. This gives an indication of the effect of the specific NO in a working system, however the translational ability of this research to humans must be considered.

Additionally, the human sample that will be analysed for the candidate marker must be considered. Each sample: blood, cerebrospinal fluid (CSF) and microdialysis fluid pose challenges for methods of analysis. Broadly, a plasma or serum sample would have the added influence of systemic function, and its analytes would not be directly cerebral. Arterio-jugular venous differences have been suggested as a means of determining the net production of mediators across the cerebrovascular bed, however, the small absolute difference between the samples makes identification of a consistent difference difficult.^135^ Ventricular CSF is valuable, as it is central nervous system-specific, but in general is not readily accessible in TBI patients as the use of extra-ventricular drains (EVDs) as part of clinical management depends on the local practice and varies between countries and trauma centres. Microdialysis monitoring has been shown to be useful in clinical practice because it offers insight into brain metabolism. In addition, it samples analytes directly from the extracellular fluid and hence may offer insight into processes occurring directly in the brain. However, the analysis of microdialysate is challenging as the catheter membrane filter only allows retrieval of substances with a molecular weight less than 100 kDa. Furthermore, the sample volume yield is low which often requires pooling of samples for meaningful analysis, depending on the analyte(s). This then decreases the temporal information we can investigate, regarding the patient’s inflammatory profile.

An enzyme-linked immunosorbent assay (ELISA) would be ideal for the quantification of candidate markers in the blood, CSF or microdialysate of human patients as it is relatively quick, affordable, and easy to use. The detection range, and degree of specificity varies from plate to plate, however a high sensitivity and specificity is generally considered an advantage of this method. Furthermore, a multiplexing method (such as Luminex®), would allow for the simultaneous investigation of multiple potential biomarkers along a candidate pathway – where the analysis of upstream activation markers, intermediate effector markers, as well as terminal or downstream candidates can be explored in one sample run. This would provide additional information about the pathway, such as extent of activation in pathology. However, ELISA and Luminex assays are offline techniques that require several hours’ analysis time in a laboratory with specialised equipment, so cannot be used for assessing patients in near-real-time that would be necessary for implementing individualised therapy. Thus, point-of-care rapid assays would be highly desirable, with potential for patient benefit.

## Assessing cerebral autoregulation in humans

Human CA assessment is difficult, partly because it is a concept and not a directly quantifiable phenomenon.^136^ Assessment of autoregulation is traditionally classified into two methods - static and dynamic testing. Loosely speaking, this division is dependent on whether assessment is based on the degree of suppression of the blood pressure driven changes in cerebral blood flow, or the speed of engagement of that mechanism. Static readings can be achieved by measuring changes in CBF (or CBFV, cerebral blood flow velocity) in response to induced step changes in MAP (measurements taken before and after the change in MAP has stabilised). These can be obtained via the administration of medication or a shift in blood volume.^136^ Static testing provides direct information about the range of CPP in which CA is active and can be used to recreate the classic autoregulation curve.^137^ Dynamic CA testing, on the other hand, refers to the rapid phase of changes in MAP, where the CBF (or surrogate thereof) response time gives us an indication of CA.^138,139^ As this method of CA assessment does not require a new steady state to be achieved (thus allowing rapid and readily repeated assessment), it may be more clinically useful. MAP pressure changes can be induced or spontaneous, with induced changes including the thigh cuff release,^140^ Valsalva manoeuvre,^141^ tilt test^142^ or transient hyperaemic response test.^143^ Assessing cerebral autoregulation with spontaneous fluctuations in MAP can be done in the time and frequency domain. Spontaneous MAP fluctuations are inherently non-stimulatory and therefore provide autoregulatory information without the additional risks of static testing, which makes it applicable to a wider patient population.^144^

Most accessible CA testing involves a surrogate measure of CBF or cerebral blood volume (CBV) changes, which can be invasive or non-invasive. Invasive surrogates include brain tissue oxygenation monitoring (CBF surrogate), and ICP monitoring (CBV surrogate), where signals from these monitoring methods can be correlated with MAP, and the resulting correlation coefficient provides autoregulatory information.^20^ PRx, the correlation index between MAP and ICP, is the most widely used index of pressure reactivity and can be calculated continuously and in real time at the patient bedside.^20^ The current gold-standard of non-invasive CBF measurement is the transcranial Doppler (TCD)-measured CBFV, with Mx being the correlation coefficient between MAP and CBFV.^140,145^ The use of Near Infrared Spectroscopy (NIRS) in non-invasive CA assessment is gaining popularity as it may provide an opportunity for easy, long-term continuous monitoring.^146^

It is of great importance to consider the method of CA assessment when attempting to relate CA status and the inflammatory cascade. Biomarker levels may have better associations with certain CA indices and not others, and in order to identify key molecular pathways involved in CA disturbance, methods of assessment need to be considered. Furthermore, the clinical impact of individualised management of blood pressure, or cerebral perfusion pressure based on the continuous monitoring of CA,^147^ is likely to be much improved if augmented by better understanding of those pathways.

## Applicability and transferability to other pathological states in humans

CBF disturbance and the loss of CA is a common cascade following several neurological pathologies such as stoke,^148,149^ meningitis,^150,151^ and intracerebral haemorrhage,^152,153^ as well as non-neurological originating pathologies like metabolic disease.^27^ Hence, work on identifying the molecular pathways underpinning CA and CBF control, and how derangements in these pathways manifest clinically, has wider applicability and treatment potential.

While each disease manifests in a unique way in every patient, the molecular underpinnings of this common pathway and CBF control may be maintained across diseases. This primary research into vascular inflammation and mediators of CBF control could identify the underlying molecular pathways, and targets to control these pathways will work towards avoiding and/or ameliorating CA dysfunction in the clinical setting. This may have a positive effect on mortality and outcome for many pathological states and neurological diseases. While the primary pathways may be common for these pathologies where CA is disturbed, it is important to note that the pathological mechanisms (especially of primary and secondary brain injury) and consequences of proinflammatory states differ. Hence, it is likely that specific biomarkers will be elevated in some states, and not others. Therefore, fundamental explorations of CBF control may be applicable and transferable, but development of specific biomarkers indicative of CA status of the patient, may need more fine-tuning to the disease, mechanism of pathology, and the individual.

## Conclusion: comments on future work and clinical utility

Endothelial dysfunction and vascular inflammation offer a plausible link to CA disturbances following both SAH and TBI, and, in principle, a promising avenue for therapy. This review has discussed candidate biomarkers that provide the highest yield inflammatory cascades involved in CBF maintenance. While many of these pathways overlap and influence each other, candidate markers of these pathways may offer insight into the molecular underpinnings of CBF disturbances in brain injury. Clinical trials, such as the ongoing investigations into optimal CPP targets, could incorporate the measurement of suggested candidate markers. In doing so, such studies could connect candidate inflammatory pathways with CBF disturbances, and addressing these disturbances may improve treatment of acute brain injury patients.

The easy and robust measurement of these candidate markers will depend on many factors, and future work could aim at exploring these pathways to identify the most influential ones in CA. Considering the difficulties of measuring NO derived from the individual isoforms of NOS, the complement pathway (particularly lectin activation) may offer the most promise.

In conclusion, this review describes candidate biomarkers of CA and mechanisms at which future work can be directed. Once the molecular underpinnings of CBF maintenance have been identified, further studies into targeting these pathways, and hence attenuating or possibly preventing CA impairment after acute injury can be conducted, with the potential to improve patient management and outcome.
